# Investigations of nitazoxanide molecular targets and pathways for the treatment of hepatocellular carcinoma using network pharmacology and molecular docking

**DOI:** 10.3389/fphar.2022.968148

**Published:** 2022-07-25

**Authors:** Shakeel Ahmad Khan, Terence Kin Wah Lee

**Affiliations:** ^1^ Department of Applied Biology and Chemical Technology, The Hong Kong Polytechnic University, Kowloon, Hong Kong SAR, China; ^2^ State Key Laboratory of Chemical Biology and Drug Discovery, The Hong Kong Polytechnic University, Kowloon, Hong Kong SAR, China

**Keywords:** nitazoxanide, network, pharmacology, molecular docking, hepatocellular carcinoma

## Abstract

Nitazoxanide has been investigated for colorectal cancer and breast cancer. However, its molecular targets and pathways have not yet been explored for hepatocellular carcinoma (HCC) treatment. Utilizing a network pharmacology approach, nitazoxanide’s potential targets and molecular pathways for HCC treatment were investigated. HCC targets were extracted from the GeneCards database. Potential targets of nitazoxanide were predicted using Swiss Target Prediction and Super Pred. Intersecting targets were analyzed with VENNY online tool. Using Cytoscape, a protein-protein interaction (PPI), cluster, and core targets-pathways networks were constructed. Using the Database for Annotation, Visualization and Integrated Discovery (DAVID), gene ontology (GO), and Kyoto Encyclopedia of Genes and Genomes (KEGG) pathway enrichment analyses were conducted. The nitazoxanide was molecularly docked with anti-HCC core targets by employing Auto Dock Vina. A total of 168 potential targets of nitazoxanide, 13,415 HCC-related targets, and 153 intersecting targets were identified. The top eight anti-HCC core targets were identified: SRC, EGFR, CASP3, MMP9, mTOR, HIF1A, ERBB2, and PPARG. GO enrichment analysis showed that nitazoxanide might have anti-HCC effects by affecting gene targets involved in multiple biological processes (BP) (protein phosphorylation, transmembrane receptor protein tyrosine kinase (RTKs) signaling pathway, positive regulation of MAP kinase activity, etc.). KEGG pathways and core targets-pathways network analysis indicated that pathways in cancer and proteoglycans in cancer are two key pathways that significantly contribute to the anti-HCC effects of nitazoxanide. Results of molecular docking demonstrated the potential for active interaction between the top eight anti-HCC core targets and nitazoxanide. Our research offers a theoretical basis for the notion that nitazoxanide may have distinct therapeutic effects in HCC, and the identified pharmacological targets and pathways might function as biomarkers for HCC therapy.

## Introduction

HCC is a kind of cancer that often affects people who have a history of hepatitis or cirrhosis. Owing to increasing malignancy and morbidity, it is the second leading cause of global cancer-related demise (Khan and Lee, 2022; Yang et al., 2022). Several efficient therapeutic approaches, including biological therapy, interventional radiology, chemotherapy, resection, tumor ablation, transcatheter arterial chemical embolization (TACE), liver transplantation, etc., have been extensively employed in treating HCC in recent decades (Stehlin et al., 1988; El-Serag, 2011; Balogh et al., 2016; Hilmi et al., 2020). HCC detection in patients at an early stage is critical and has significant importance because it is strongly linked to a patient’s prognosis since interventional therapy delivered at a preliminary phase of HCC may significantly improve patient outcomes. Regrettably, patients are often identified with HCC at an intermediate or advanced stage, precluding resection and transplantation. Blood vessels’ active invasion, resulting in extrahepatic and intrahepatic metastases, is linked to a poor prognosis after surgical or medical treatment because of a high recurrence rate (Dutta and Mahato, 2017; Mohs et al., 2017; Daher et al., 2018).

Moreover, chemotherapy treatment with sorafenib (tyrosine multi-kinase inhibitor) has also been identified as a promising therapeutic for an advanced stage of HCC. However, its treatment can only increase overall survival by about 3 months (Llovet et al., 2008; Yuan et al., 2022). On the contrary, numerous tyrosine multi-kinase inhibitors have been shown to be dangerous or to have no effect on patient survival (Cainap et al., 2015). Efforts have been undertaken to develop promising pharmacological solutions against HCC in the lack of adequate preventative or treatment methods in order to provide patients with alternative treatment choices and enhance patient life expectancies (Jindal et al., 2019).

In this instance, scientists are trying very hard to find effective therapeutics for the treatment of HCC by adopting a drug repurposing strategy compared to traditional drug designing and development owing to its several limitations, including failure in expensive late-stage clinical trials, high attrition rates, takes a long time, high cost, etc. (Pfab et al., 2021). Repurposing currently utilized drugs offers several advantages over developing an entirely new therapeutic (Zamboni et al., 2012; Gupta et al., 2013). In a drug repurposing strategy, the failure risk is lower in terms of safety, toxicity, and formulation information already available, therefore drastically reducing the costs necessary to get the medications to patients. In fact, bringing a repurposed drug to market is ten times less expensive than bringing a unique chemical molecule to market. Since data from clinical trials about pharmacokinetics, bioavailability, etc., is already available, the drug repurposing strategy also reduces the time to make a new drug (Zamboni et al., 2012; Gupta et al., 2013; Nosengo, 2016; Pushpakom et al., 2019; Pfab et al., 2021).

Nitazoxanide was first developed as an anthelmintic agent and is an antimicrobial agent authorized by the FDA (Sisson et al., 2002; Pfab et al., 2021). Müller et al. repurposed nitazoxanide for colorectal cancer (CRC), and it has shown anticancer activity by inhibiting apoptosis, DNA fragmentation, nuclear condensation, and cell proliferation. It specifically targeted glutathione-S-transferase P1 (GSTP1), activating the AMPK pathway while suppressing c-Myc, mTOR, and WNT signaling in CRC (Müller et al., 2008; Senkowski et al., 2015). Moreover, nitazoxanide has been reported to suppress c-Myc expression, leading to tumor growth suppression and apoptosis induction in breast cancer (Fan-Minogue et al., 2013). Nitazoxanide has not been explored for HCC treatment and could be expected to down regulate the overexpressed proteins implicated in the proliferation of HCC malignancy.

**FIGURE 1:**
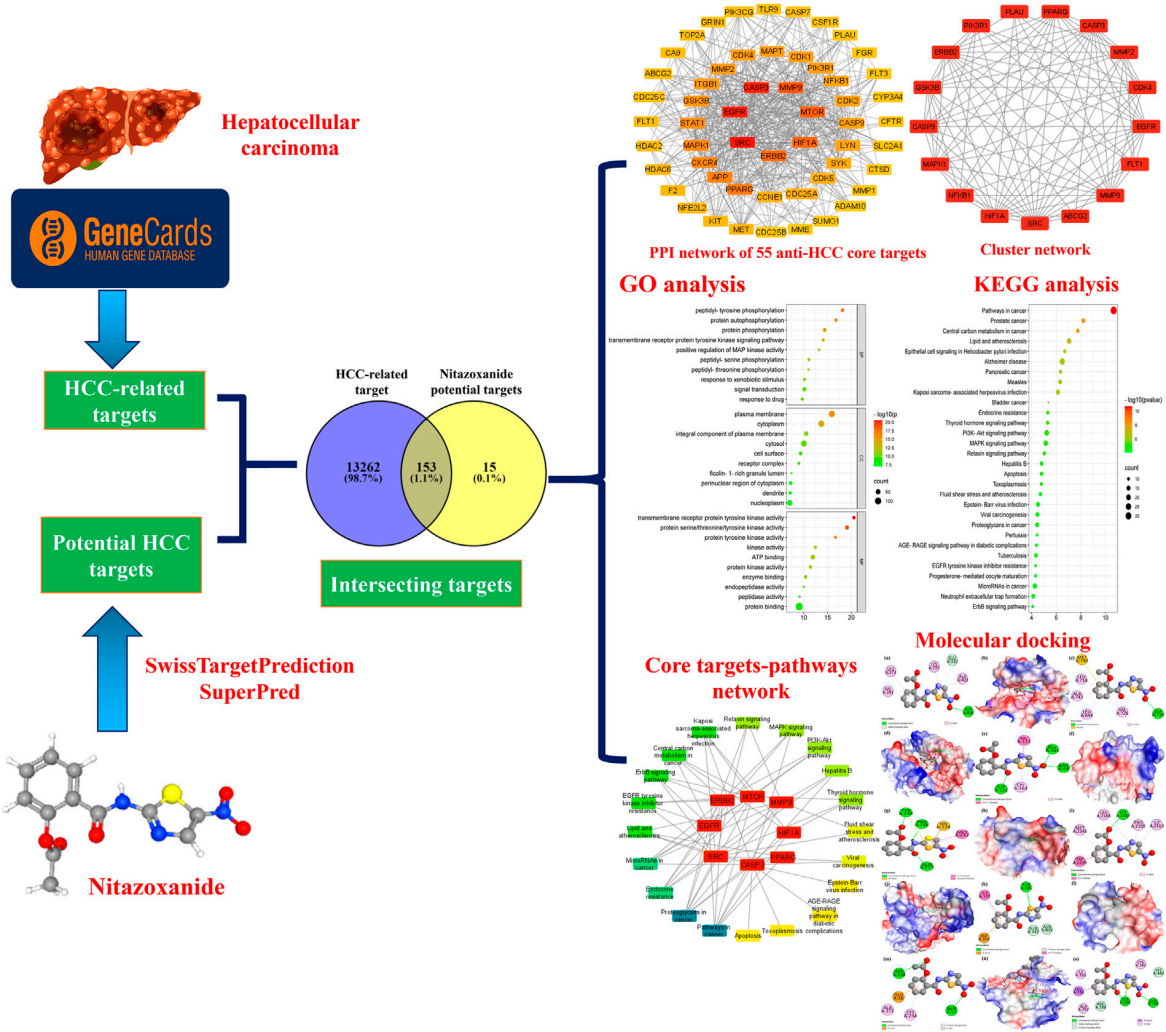
The flow chart of this research.

Network pharmacology, multi-omics data, molecular docking, and public medical databases have enabled an alternative computational drug discovery approach and are extensively used to design and develop therapeutic drugs for many cancer types (Khan and Lee, 2022; Yuan et al., 2022). Computational drug repurposing is particularly intriguing since it allows for quicker screening of candidate drugs than traditional drug design and development (Luo et al., 2021). Computational drug repurposing develops interactions between proteins, diseases, genes, and therapeutic candidates based on open-access databases. It suggests viable therapeutic candidates, assuming they target the same proteins in treating ailments (Keenan et al., 2018). Currently, several researchers are using them to design and develop therapeutic drug candidates and explore the molecular pathways of natural products implicated in the therapy of various ailments. Therefore, we have utilized different bioinformatics tools in this research, including network pharmacology and molecular docking, to computationally repurpose and identify nitazoxanide’s targets and molecular pathways that could be involved in treating HCC. The flow chart of this research is presented in Figure 1.

## Materials and methods

### Targets prediction of nitazoxanide

The targets of nitazoxanide were predicted using Swiss Target Prediction (http://www.swisstargetprediction.ch/, accessed on 12 May 2022) and SuperPred (https://prediction.charite.de/, accessed on 12 May 2022) web servers with limitations to “Homo sapiens” (Nickel et al., 2014; Daina et al., 2019). The targets predicted with Swiss Target Prediction and Super Pred, which have a probability greater than zero and 50%, respectively, were selected as potential targets for nitazoxanide.

### HCC-related targets determination

The HCC-related targets were identified by exploring the Gene Cards (https://www.genecards.org/, accessed on 12 May 2022) database for the terms “hepatic cancer, hepatic carcinoma, hepatocellular carcinoma, and hepatoma” (Rebhan et al., 1997).

### Intersecting targets of HCC-related and potential targets of nitazoxanide

The intersecting targets between HCC-related and potential targets of nitazoxanide were identified using the VENNY 2.1 online tool (https://bioinfogp.cnb.csic.es/tools/venny/, accessed on 12 May 2022) (Venny, 2022). These identified intersected targets were screened for further analysis.

### Protein-protein interaction analysis

The PPI analysis was performed on identified intersected targets by employing the STRING (https://string-db.org/, version 11.5, accessed on 12 May 2022) database with limitations to “Homo sapiens” and at a medium confidence score of 0.400 (von Mering et al., 2003). Moreover, their results were further explored by uploading them to Cytoscape software (version 3.9.0, Boston, MA, United States, accessed on 12 May 2022) to find the potential targets and anti-HCC core targets based on their degrees in the network (Lopes et al., 2010). Moreover, cluster network analysis was carried out using the Molecular Complex Detection (MCODE) plugin of Cytoscape (version 3.9.0) by setting the parameters as; find clusters = in the whole network, degree cutoff = 2, node score cutoff = 0.2, K-score = 0.2, and max depth = 100.

### GO and KEGG enrichment analysis

GO, and KEGG enrichment analyses were performed on identified intersected targets (determined in section 2.3) using the database for annotation, visualization, and integrated discovery (DAVID; version 6.8) (https://david.ncifcrf.gov/, accessed on 13 May 2022) (DAVID, 2022). Both analyses were performed by keeping the parameters: species, Homo sapiens; identifier, official gene symbol; gene list, list type; and remaining parameters, default values (Li et al., 2022). The results of GO enrichment analyses are comprised of three terms, including molecular functions (MF), cellular component (CC), and biological process (BP). The top 10 GO data (MF, BP, and CC) and 30 KEGG pathways were uploaded to the Bioinformatics platform (http://www.bioinformatics.com.cn/, accessed on 13 May 2022), and the results are displayed in the form of a bubble plot (Weishengxin, 2022). The enrichment of GO and pathways was deemed substantial if *p* ≤ 0.05.

### Network construction between anti-HCC core targets and pathways

The network was established between anti-HCC core targets and molecular pathways using Cytoscape software (version 3.9.0, Boston, MA, United States; accessed on 13 May 2022) to determine the intricate relationship between them in the treatment of HCC with nitazoxanide (Lopes et al., 2010).

### Expression of anti-HCC core targets

The GEPIA database (http://gepia.cancer-pku.cn/, accessed on 14 May 2022) was used to examine the expression of the top eight anti-HCC core targets (determined in section 2.4.) in liver hepatocellular carcinoma (LIHC) (GEPIA, 2022 (Gene Expression Profiling Interactive Analysis)).

### Molecular docking

The 2D chemical structure of nitazoxanide was retrieved from NCBI Pub Chem in Spatial Data File (SDF) (National Center for Biotechnology Information, 2022). 3D structure of nitazoxanide was constructed with BIOVIA Discovery Studio Visualizer 2021 and saved in PDB format (BIOVIA DS, 2016). The protein crystal structures of eight anti-HCC core targets were retrieved from Protein Data Bank (RCSB PDB: https://www.rcsb.org/search; RCSB PDB, 2022). The water molecules and ligands from protein crystal structures were extracted BIOVIA Discovery Studio Visualizer 2021. Moreover, this was employed to prepare the grid and add polar hydrogens to proteins. Each protein in PDB format was uploaded to AutoDock Vina (version 1.2.0.) and added the Kollman and Gasteiger partial charges. The PDB file of the 3D structure of nitazoxanide was then uploaded to AutoDock Vina. Proteins and nitazoxanide files were converted into pdbqt format using AutoDock Vina, and then they were utilized to write scripts for molecular docking (Trott and Olson, 2010). The docked complexes of proteins and nitazoxanide were obtained and further analyzed to determine their molecular interactions by employing BIOVIA Discovery Studio Visualizer 2021 (BIOVIA DS). Binding energy less than zero suggests that the ligand molecule may readily bind to the pockets of the targeted proteins. It is generally accepted that a lower binding energy value for a docked complex of ligand and receptor implies a stronger binding (Trott and Olson, 2010).

## Results

### Potential targets of nitazoxanide

The nitazoxanide targets were predicted using the Swiss Target Prediction and Super Pred web servers with “Homo sapiens” limitations (Nickel et al., 2014; Daina et al., 2019). A total of 168 potential targets were retrieved with a probability greater than zero and 50%.

### HCC-related targets

By exploring the GeneCards database for the terms “hepatic cancer, hepatic carcinoma, hepatocellular carcinoma, and hepatoma,” 13,415 HCC-related targets were retrieved (Rebhan et al., 1997).

### Identification of intersecting targets

**FIGURE 2:**
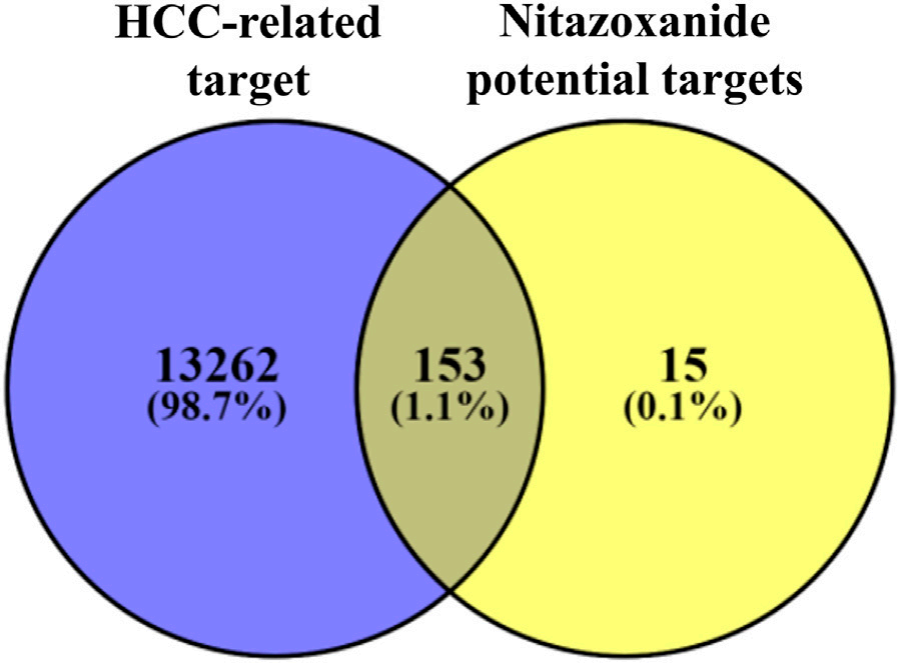
The intersecting targets between HCC-related and potential targets of nitazoxanide (HCC = Hepatocellular carcinoma).

A total of 153 intersecting targets were identified between HCC-related and potential targets of nitazoxanide using the VENNY 2.1 online tool (Venny, 2022) (Figure 2). These identified intersecting targets were deemed as potential anti-HCC targets implicated in the treatment of HCC with nitazoxanide.

### PPI network analysis

**FIGURE 3:**
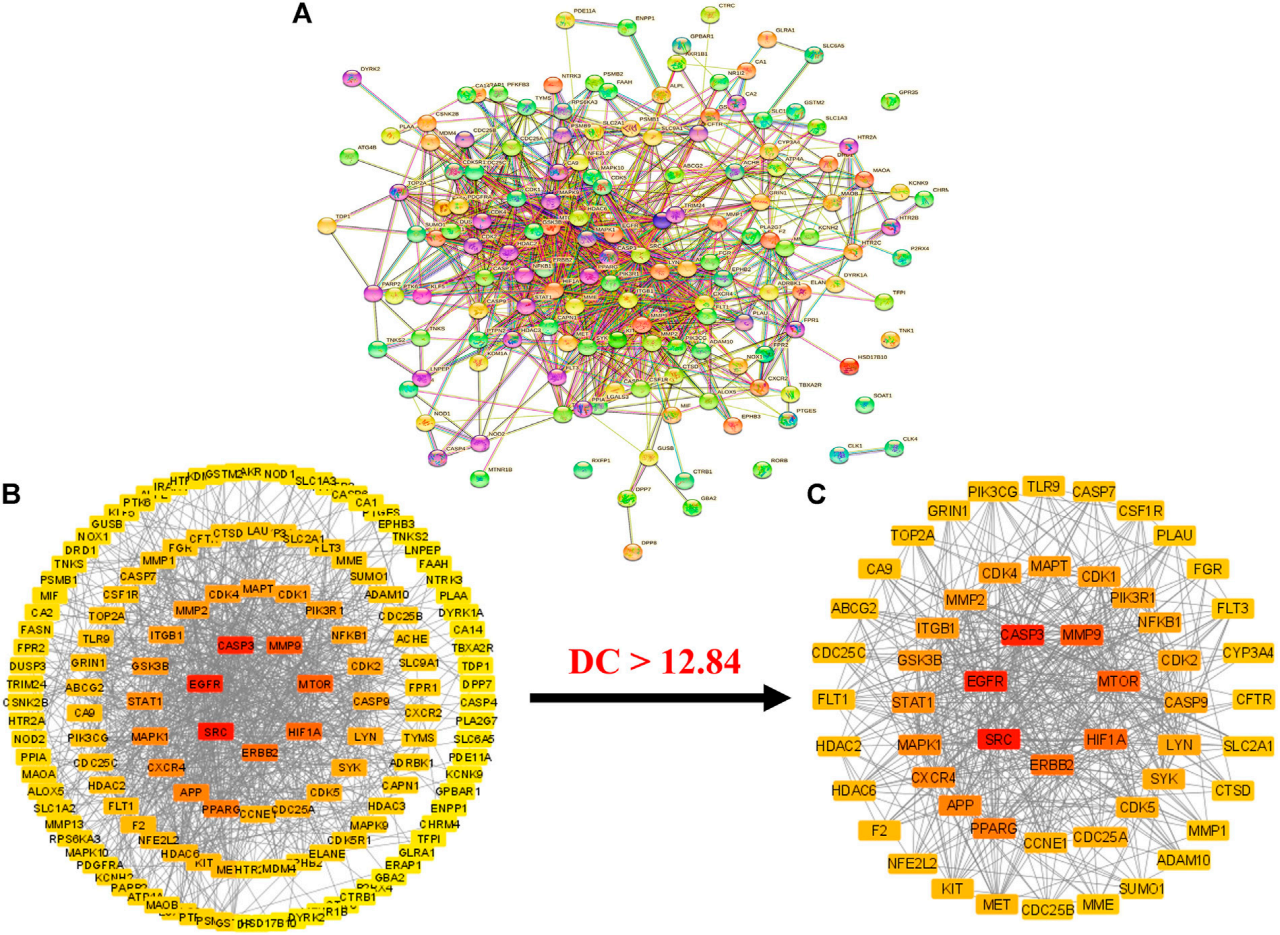
**(A)** STRIN PPI network **(B)** PPI network of intersecting targets **(C)** A hub network of 55 anti-HCC core targets. In both networks **(B,C)**, the transition from red to yellow represents the degree shift from highest to lowest for each node (PPI = Protein-protein interaction).

Intersecting targets (153) were uploaded to the STRING database with limitations to the species “Homo sapiens” (von Mering et al., 2003). A PPI network was obtained, which consisted of 153 nodes and 939 edges (Figure 3A). The average node degree in the network was 12.3. Moreover, the PPI network presented a 0.462 average local clustering coefficient and 406 expected number of edges. The STRING results of the PPI analysis were further imported to Cytoscape software (version 3.9.0) for better understanding and visualization of the network (Lopes et al., 2010).

The results demonstrated that the PPI network consists of 148 nodes (with the elimination of five disconnected nodes) and 939 edges (Figure 3B). The elimination of disconnected nodes from the network by the Cytoscape software (version 3.9.0) was also reported by Liu et al. (Liu et al., 2020). Moreover, network centralization, heterogeneity, density, diameter, and radius were 0.330, 0.937, 0.089, 6, and 3, respectively. The clustering coefficient, characteristics path length, and the average number of neighbors were 0.409, 2.439, and 12.849, respectively.

**FIGURE 4:**
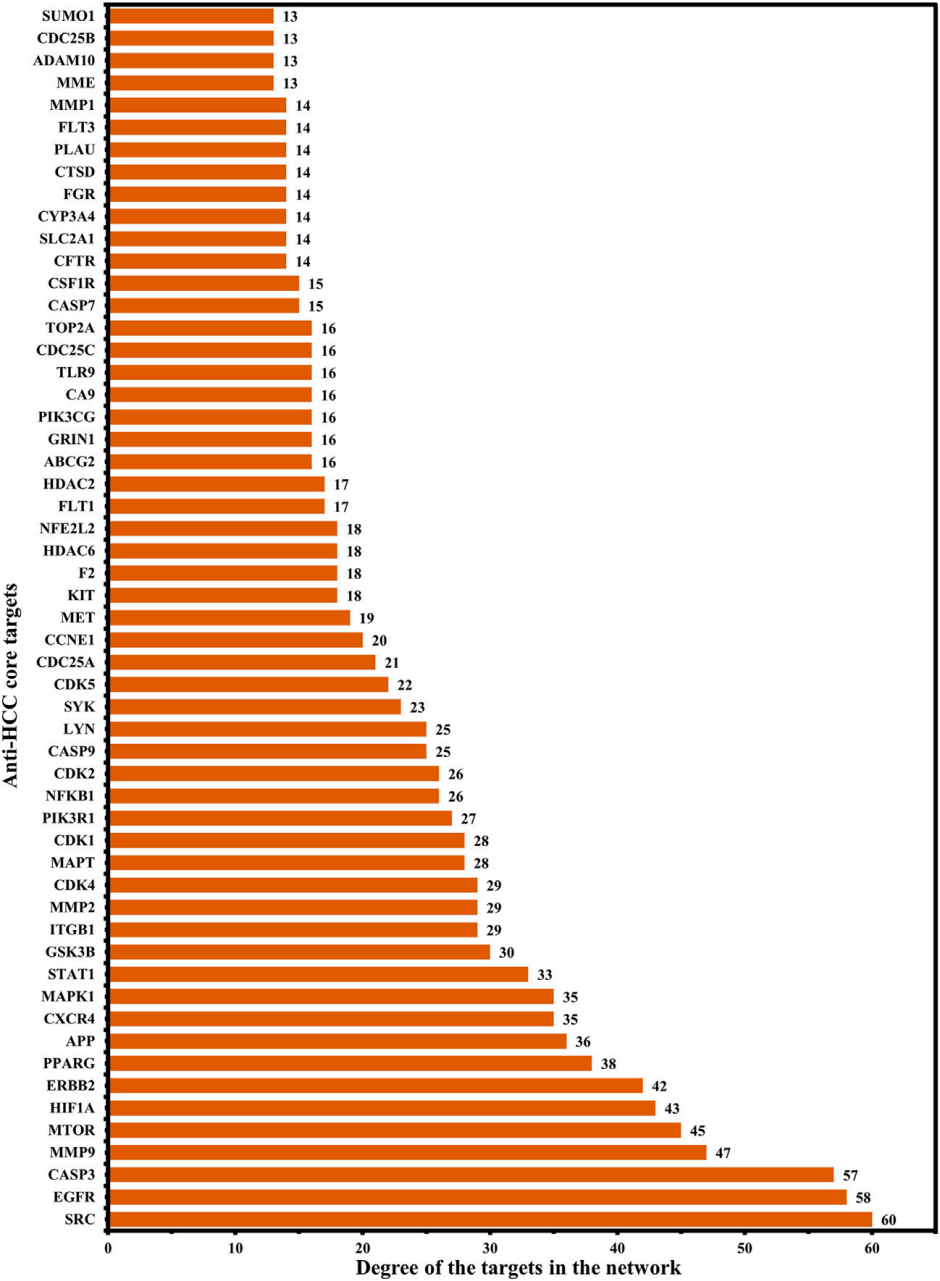
55 anti-HCC core targets in hub network ranked by DC > 12.84 (DC = Degree centrality).

Further, a hub network of targets with degrees greater than the average DC (12.84) was extracted and identified 55 potential HCC targets, which were classified as anti-HCC core targets (Figure 3C). The identified 55 anti-HCC core targets are presented in a bar graph based on their degree in the network, as shown in Figure 4. The top eight anti-HCC core targets are SRC (degree 60), EGFR (degree 58), CASP3 (degree 57), MMP9 (degree 47), mTOR (degree 45), HIF1A (degree 43), ERBB2 (degree 42), and PPARG (degree 38). These eight anti-HCC core targets were further investigated for molecular docking analysis with nitazoxanide.

### Clusters network analysis

**FIGURE 5:**
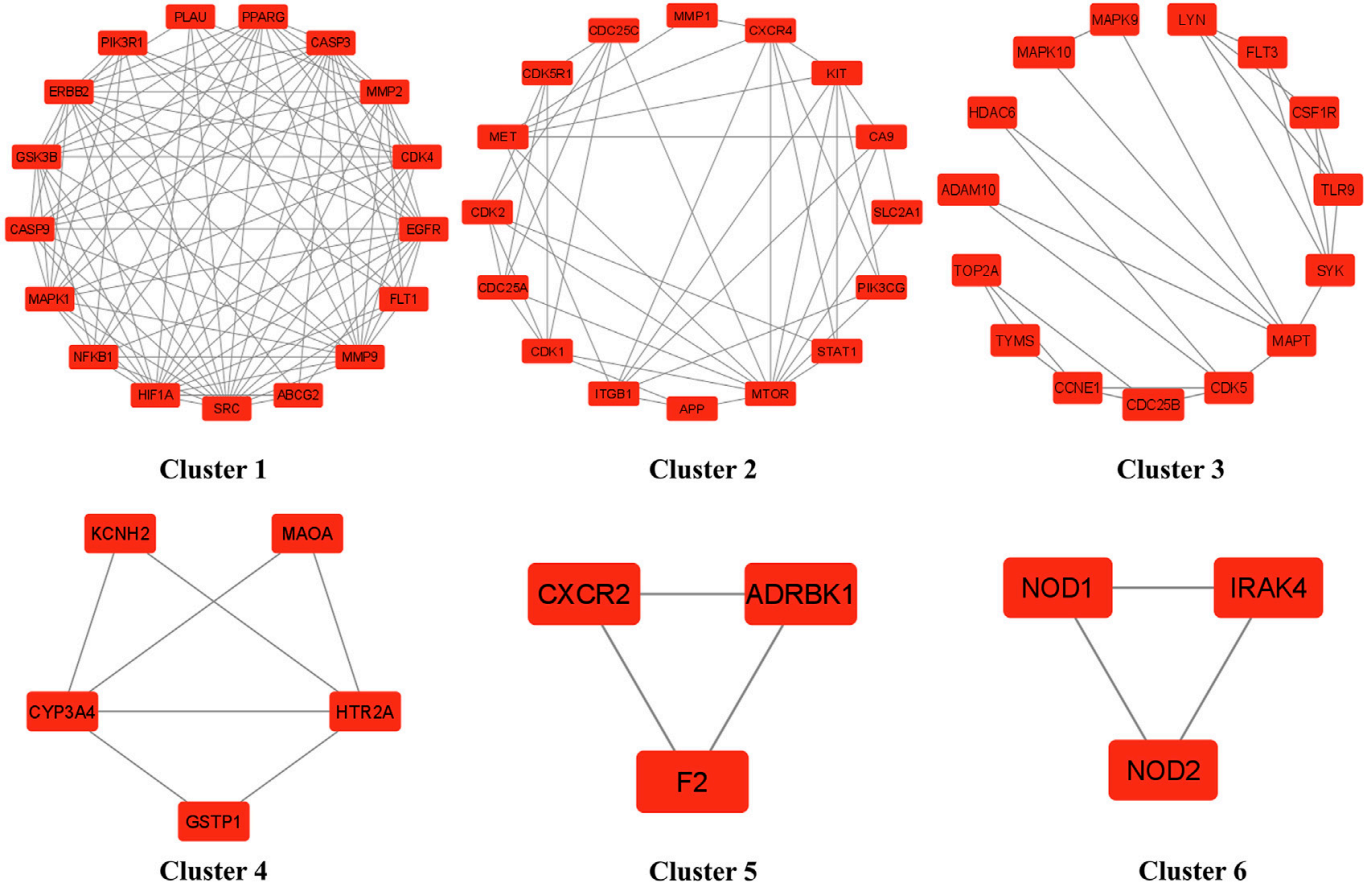
Six clusters of PPI network (PPI = Protein-protein interaction).

The cluster network analysis was further carried out on the constructed PPI network (Figure 3B) using the Molecular Complex Detection (MCODE) plugin of Cytoscape software (version 3.9.0). The PPI network was clustered into six clusters, as shown in Figure 5. Clusters 1, 2, 3, and four have 17 nodes and 97 edges, 16 nodes and 42 edges, 15 nodes and 25 edges, and five nodes and seven edges, respectively. While clusters five and six have three nodes and three edges. All of these clusters indicated the existence of potential HCC-targets for the nitazoxanide drug’s therapeutic effects. Moreover, clusters one and two show the presence of the top eight anti-HCC core targets identified in section 3.4 (Figures 3C, 4). Cluster 1 has seven out of eight anti-HCC core targets: SRC, EGFR, CASP3, MMP9, HIF1A, ERBB2, and PPARG. On the other hand, cluster 2 has one out of eight anti-HCC core targets such as mTOR. Hence, cluster network analysis corroborated the findings of the hub network.

### Expression of anti-HCC core targets in LIHC

**FIGURE 6:**
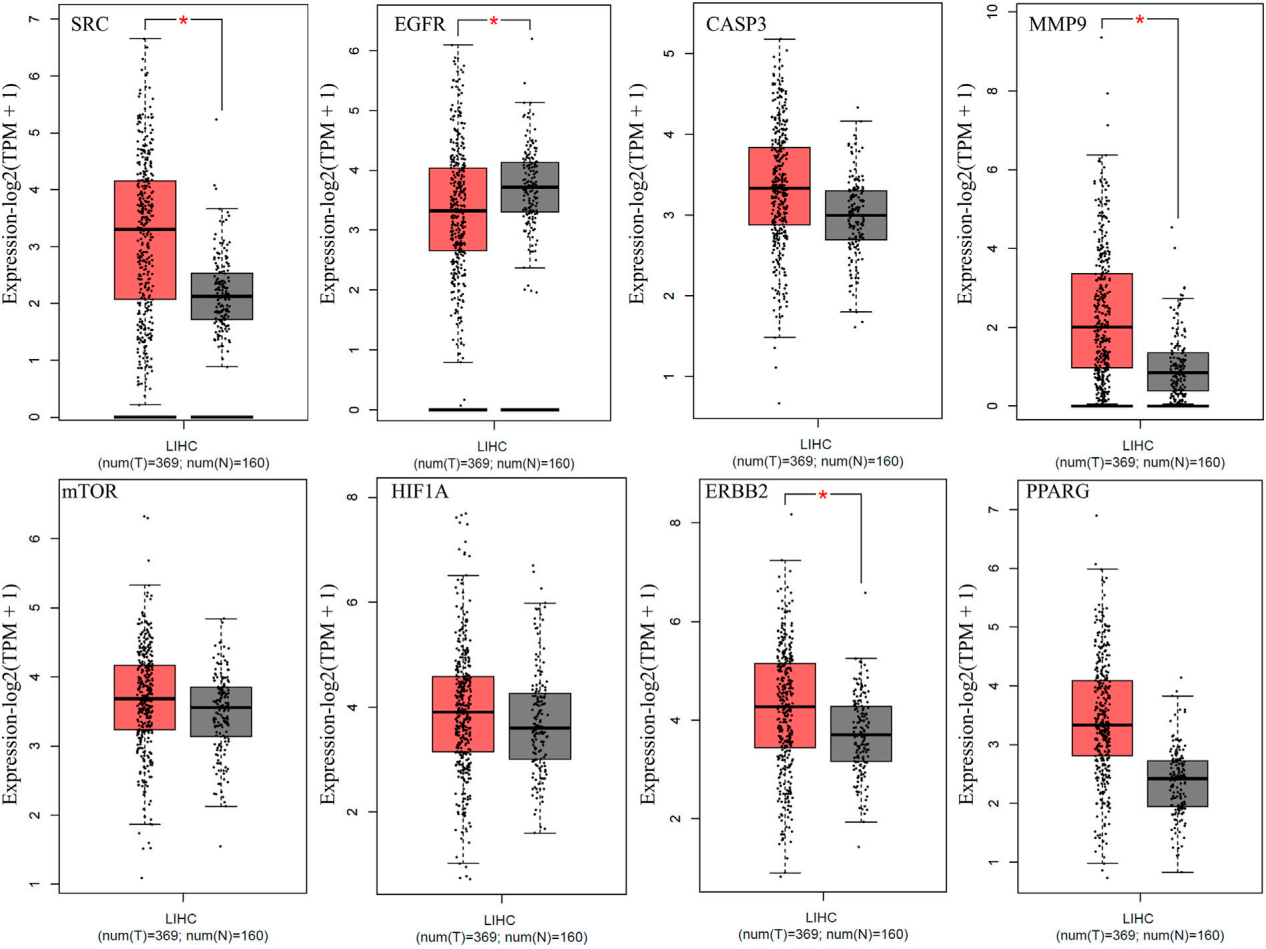
Expression of top eight anti-HCC core targets in LIHC (Red and grey colored boxes represent tumor and normal cells, respectively) (LIHC = Liver hepatocellular carcinoma).

The expression of the top eight anti-HCC core targets (SRC, EGFR, CASP3, MMP9, mTOR, HIF1A, ERBB2, and PPARG) in LIHC and normal samples were analyzed using the GEPIA database. The analysis results demonstrated that anti-HCC core targets were differentially expressed in LIHC and normal samples (Figure 6). These results corroborated that these eight anti-HCC core targets are strongly correlated to the development and progression of LIHC.

### GO enrichment analysis

**FIGURE 7:**
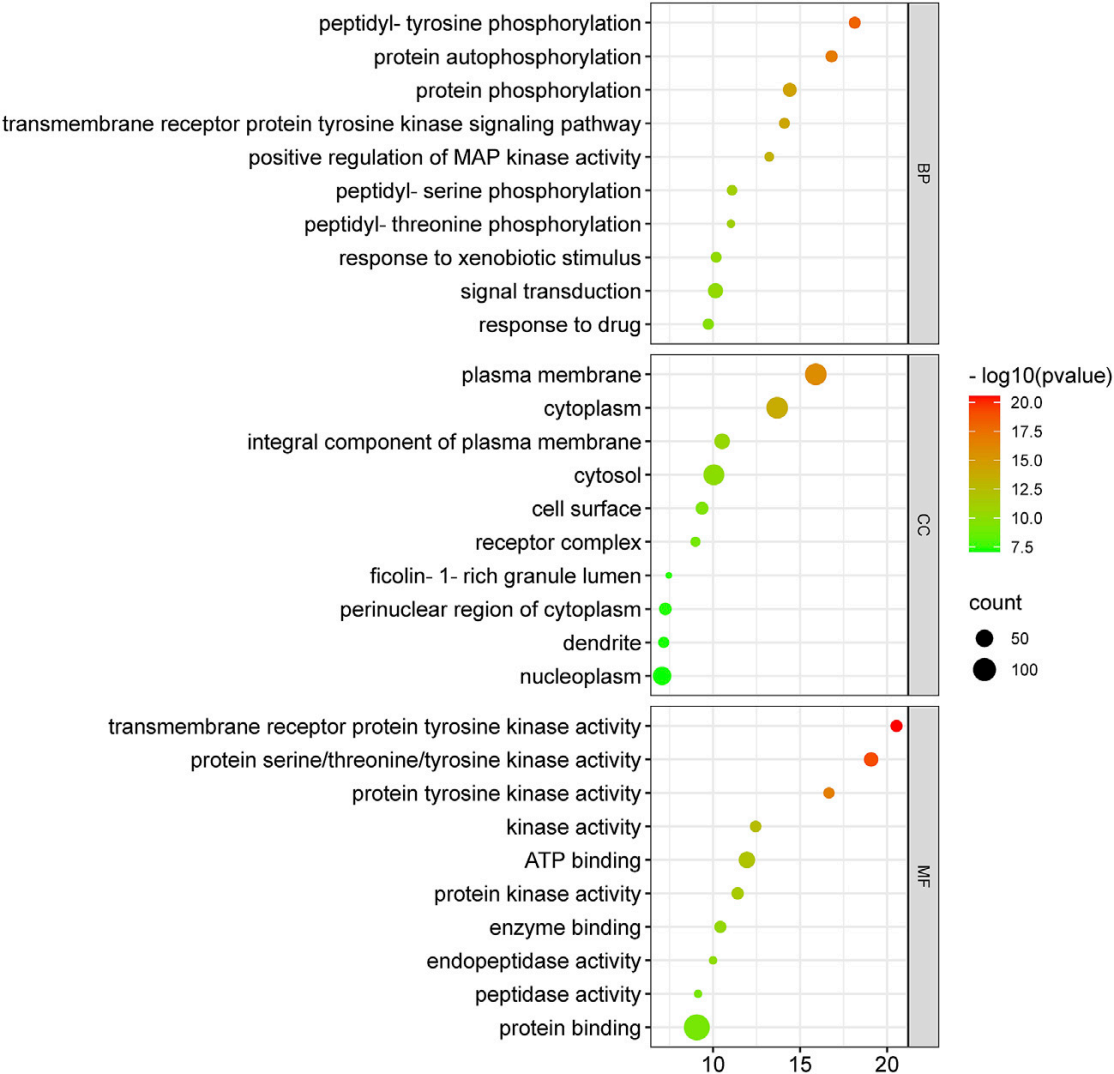
Top 10 GO enriched analysis of 153 intersecting targets involved in anti-HCC effects of nitazoxanide drug (GO = Gene ontology).

Anti-HCC effects of nitazoxanide drug were further investigated by performing GO enrichment analysis on 153 intersecting targets. The top 10 enriched GO terms (BP, MF, and CC) were identified. The results are presented in Figure 7. The targets attributed to the anti-HCC effects of nitazoxanide drug are implicated in multiple BP, which include peptidyl-tyrosine phosphorylation, protein autophosphorylation, protein phosphorylation, transmembrane receptor protein tyrosine kinase signaling pathway, positive regulation of MAP kinase activity, peptidyl-serine phosphorylation, etc. On the other hand, the targets implicated in the treatment of HCC with nitazoxanide drug are involved in multiple CC, including the plasma membrane, cytoplasm, integral components of the plasma membrane, cytosol, cell surface, etc. Moreover, results demonstrated that targets by which nitazoxanide treats HCC are implicated in multiple MF such as transmembrane receptor protein tyrosine kinase activity, proteins serine/threonine/tyrosine kinase activity, protein tyrosine kinase activity, kinase activity, ATP binding, etc.

### KEGG enrichment analysis

**FIGURE 8:**
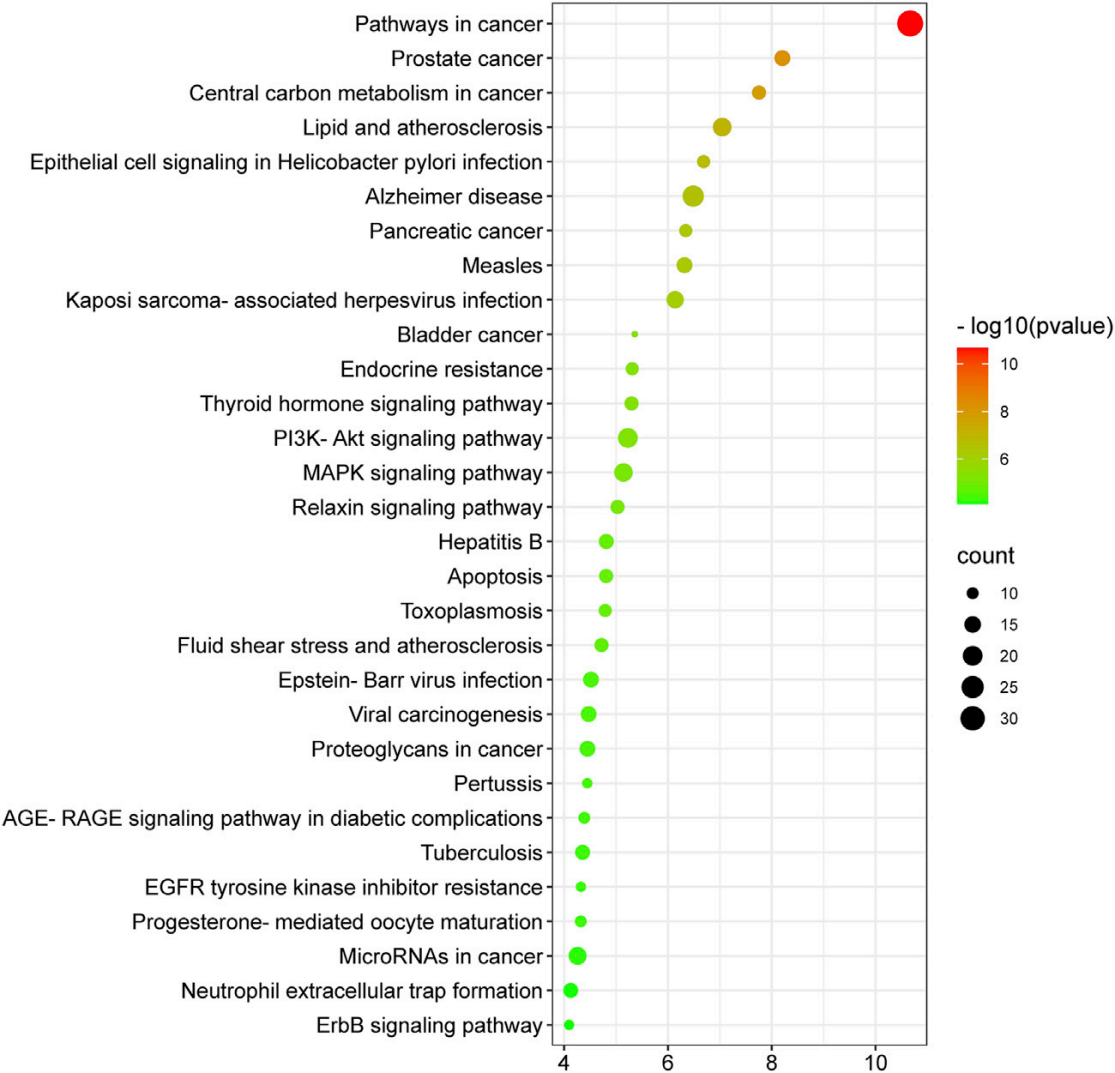
Top thirty KEGG enriched pathways of 153 intersecting targets involved in anti-HCC effects of nitazoxanide drug.

The molecular mechanisms involved in the anti-HCC effects of nitazoxanide drug were further investigated by performing a KEGG pathway enrichment analysis on 153 intersecting targets. A total of 78 enriched KEGG pathways were identified at *p* ≤ 0.05. The top 30 KEGG pathways were presented in Figure 8 in a bubble plot form. The molecular mechanism attributed to the anti-HCC effects of nitazoxanide drug might be involved in pathways in cancer, PI3K-Akt signaling pathway, MAPK signaling pathway, proteoglycans in cancer, EGFR tyrosine kinase inhibitor resistance, apoptosis, hepatitis B, ErbB signaling pathway, microRNAs in cancer, etc. These findings suggest that all of these mechanisms may be implicated in a synergistic manner in the modulation of HCC by the nitazoxanide drug.

### Network between anti-HCC core targets and pathways

**FIGURE 9:**
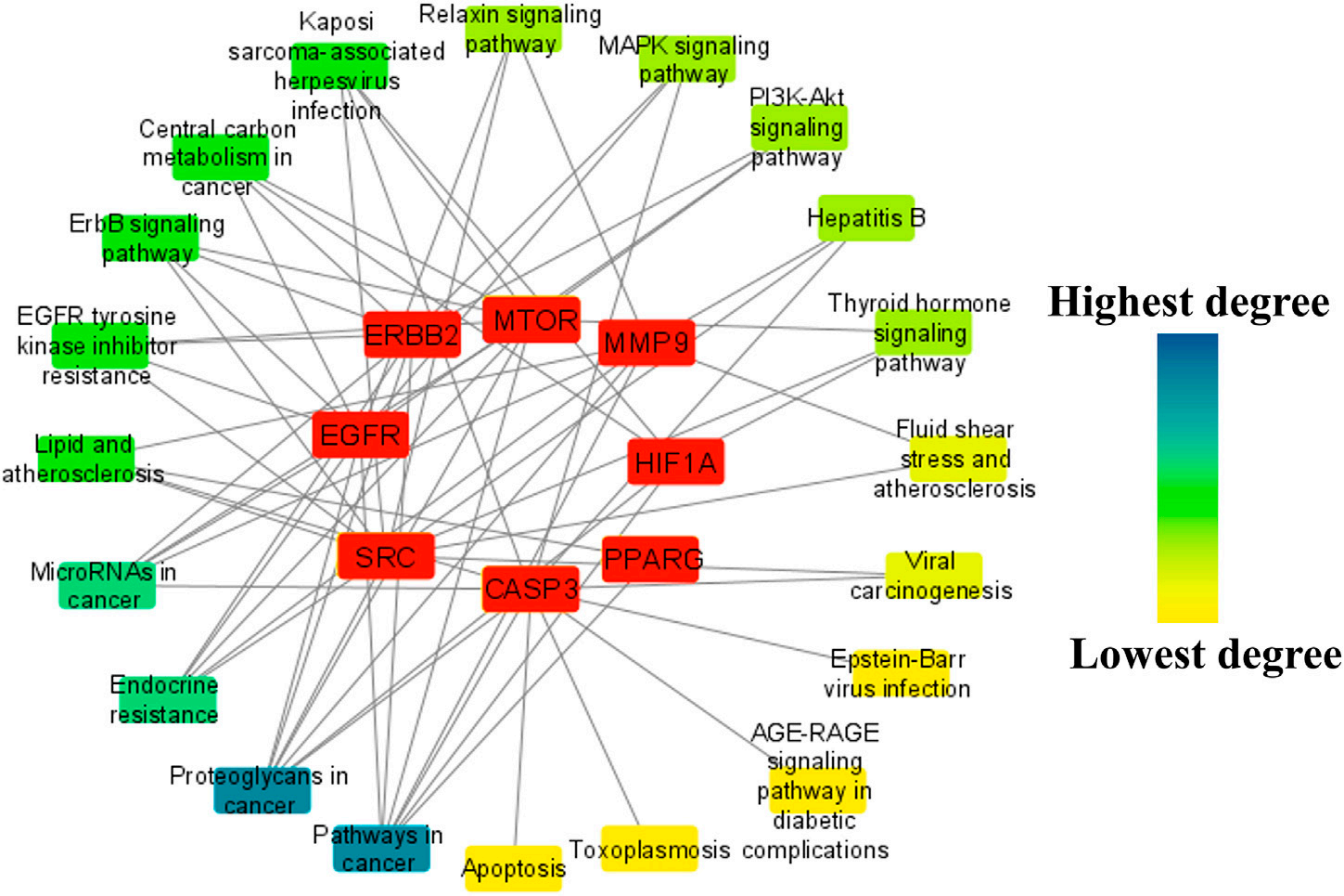
The network between the top eight anti-HCC core targets and their corresponding molecular pathways.

To identify the major pathways involved in the anti-HCC effects of the nitazoxanide drug, a network between the top eight anti-HCC core targets and their corresponding pathways were constructed. The network results demonstrated that seven anti-HCC core targets (CASP3, EGFR, ERBB2, mTOR, MMP9, HIF1A, and PPARG) followed the pathways in cancer (degree 7). On the other hand, SRC, CASP3, EGFR, ERBB2, mTOR, MMP9, and HIF1A followed the proteoglycans in cancer (degree 7) (Figure 9).

**FIGURE 10:**
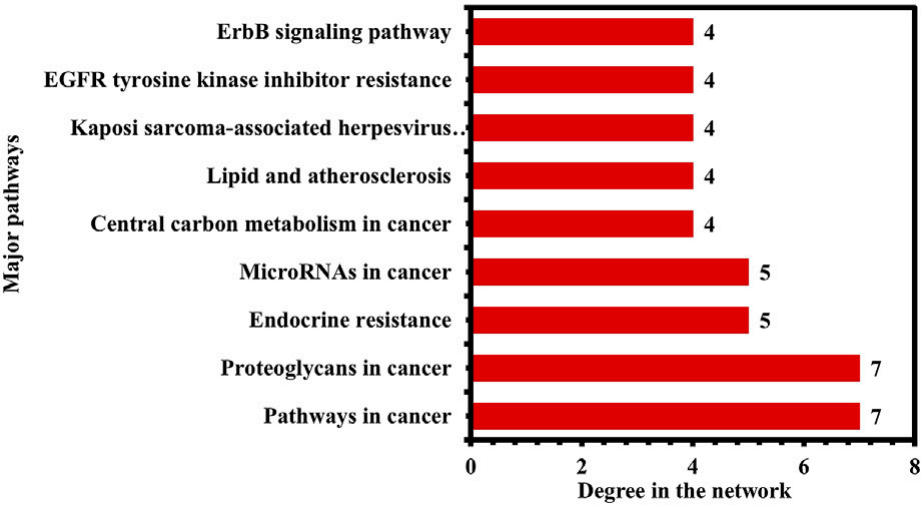
Nine pathways in the network ranked by DC > 3.35 (DC = Degree centrality).

Moreover, the pathways were further ranked by DC greater than the average DC (3.35) in the network to find the major pathways. A total of nine major pathways were identified and presented in a bar graph, as shown in Figure 10. Thus, these nine pathways may significantly contribute to the anti-HCC effects of the nitazoxanide drug by modulating the expression of anti-HCC core targets.

### Molecular docking

**FIGURE 11:**
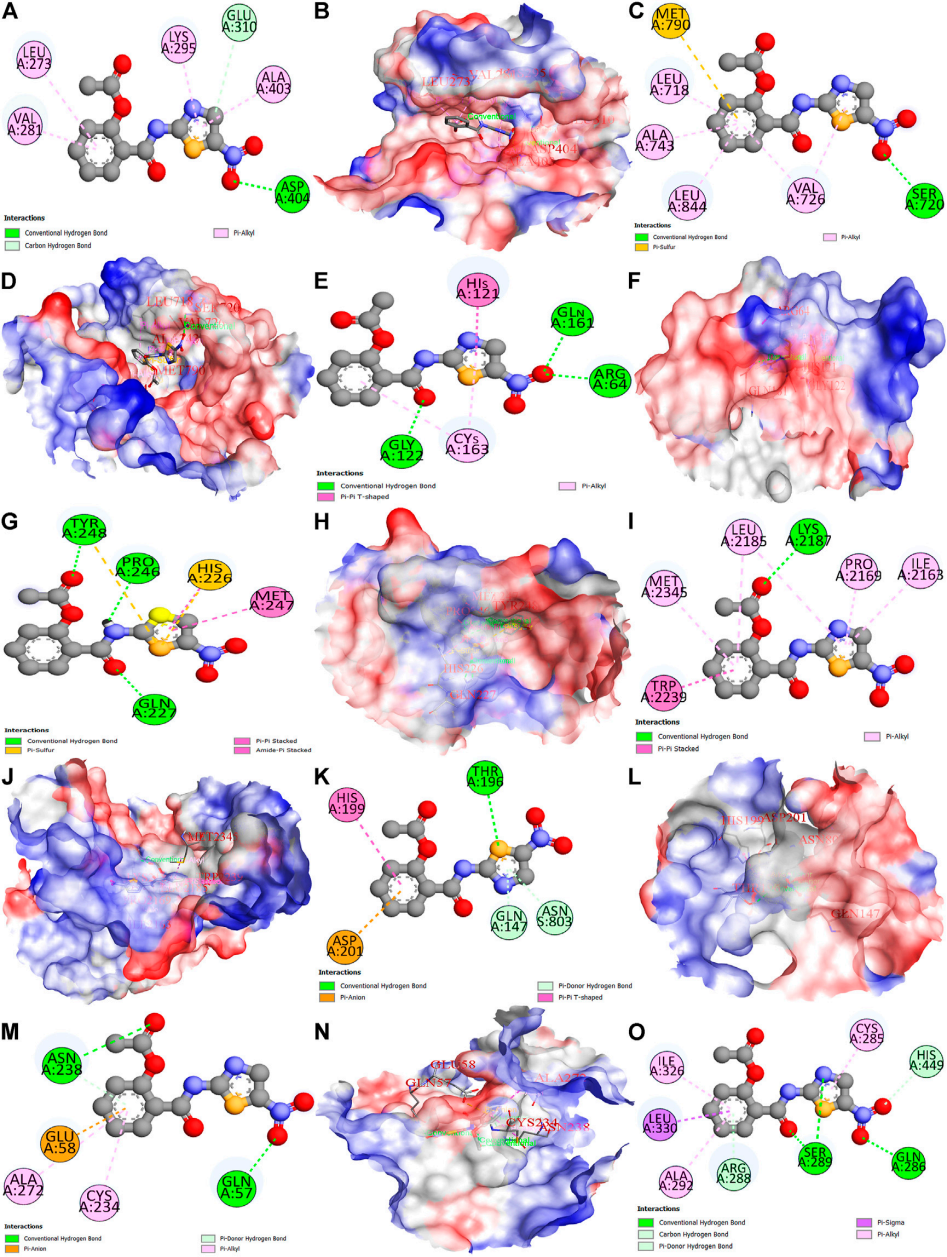
Molecular docking results of top eight anti-HCC core targets with nitazoxanide drug. Nitazoxanide drug binds with SRC (2D and 3D) **(A,B)**, EGFR (2D and 3D) **(C,D)**, CASP3 (2D and 3D) **(E,F)**, MMP9 (2D and 3D) **(G,H)**, mTOR (2D and 3D) **(I,J)**, HIF1A (2D and 3D) **(K,L)**, ERBB2 (2D and 3D) **(M,N)**, and PPARG (2D) **(O)**.

The nitazoxanide drug was molecularly docked with the top eight anti-HCC core targets (SRC, EGFR, CASP3, MMP9, mTOR, HIF1A, ERBB2, and PPARG), and the findings are shown in Table 1. Figures 11A–O depicts docked complexes of the nitazoxanide drug and anti-HCC core targets. The results revealed that the nitazoxanide drug had a high affinity for all anti-HCC core targets. However, nitazoxanide drug had a greater binding affinity with three anti-HCC core targets (SRC, MMP9, and PPARG) and had an energy score ≥ −7.0. On the other hand, the nitazoxanide drug had a high binding affinity for mTOR, EGFR, and CASP3. Furthermore, the nitazoxanide drug had a modest binding affinity for HIF1A and ERBB2, yielding an energy score of −5.1.

**TABLE 1:** Molecular docking of nitazoxanide with the top eight anti-HCC core targets.

Drug	Binding affinity (kcal/mol)							
	SRC	EGFR	CASP3	MMP9	mTOR	HIF1A	ERBB2	PPARG
Nitazoxanide	−7.0	−5.8	−5.5	−7.9	−6.9	−5.1	−5.1	−7.4

## Discussion

HCC often afflicts individuals with a history of hepatitis or cirrhosis. Owing to increasing malignancy and morbidity, it is the second leading cause of global cancer-related demise (Khan and Lee, 2022; Yang et al., 2022). In the lack of viable HCC preventive or therapeutic interventions, emerging trends have shifted toward drug repurposing instead of conventional drug discovery and development due to the latter’s many constraints (Pfab et al., 2021). The emergence of the big data era and the growth of bioinformatics approaches provide tremendous assistance for drug discovery *via* network pharmacology (Vetrivel et al., 2021). The core principle of network pharmacology is that prospective targets may be predicted by looking at their biological pathways from a network perspective. This may aid in discovering novel active medications from medicinal compounds (Zhu et al., 2019; Vetrivel et al., 2021). In this study, a network pharmacology approach was utilized to evaluate the therapeutic mechanism of nitazoxanide as a treatment for HCC. For the first time, nitazoxanide’s pharmacological effects on HCC have been examined utilizing network pharmacology and molecular docking simulations. Therapeutic targets of nitazoxanide against HCC were predicted using online databases. A total of 168 potential therapeutic targets were identified. A total of 13,415 HCC-related targets were retrieved from the online database. Furthermore, 153 intersecting targets were identified between the potential targets of nitazoxanide and HCC-related targets.

PPI and cluster network analysis of intersecting targets displayed that multiple genes such as SRC, EGFR, CASP3, MMP9, mTOR, HIF1A, ERBB2, and PPARG are implicated in the anti-HCC effects of nitazoxanide. The report demonstrates that elevated expression of SRC leads to the pathogenesis of HCC and subsequent metastasis (Zhao et al., 2015). Overexpression of EGFR has been implicated in HCC pathogenesis, and activation of this receptor contributes to HCC cells’ primary resistance to sorafenib (Sueangoen et al., 2020). Persad et al. reported the implication of CASP3 overexpression in the pathogenesis of HCC (Persad et al., 2004). Previous reports demonstrate that MMP9 is an oncogene implicated in HCC progression (Yan et al., 2013; Lu et al., 2015). Its higher expression in HCC tissues was also reported by Liu et al. (Liu B. et al., 2021). The mTOR signaling is involved in numerous cancer hallmarks such as cell growth, apoptosis suppression, etc. In HCC tissue samples, the mTOR pathway is more highly expressed than in liver cirrhotic tissue in the general vicinity (Ferrín et al., 2020). Reports show that HIF1A protein levels are considerably higher in human HCC samples and are linked with a poorer prognosis (Chen and Lou, 2017). In the last 3 decades, research indicated that ERbb2 expression is seldom associated with the development of HCC (Xian et al., 2005; Shi et al., 2019). However, alternative studies also revealed that ERBB2 expression was higher in 30–40 percent of HCC (Heinze et al., 1999; Shi et al., 2019). Moreover, GEPIA database analysis shows that all eight anti-HCC core targets were differentially expressed in LIHC and normal samples (Figure 6). Hence, based on the literature and the GEPIA database, all eight of these anti-HCC core targets played a crucial role in the progression of HCC and may be promising therapeutic targets for treating HCC with nitazoxanide.

The GO enrichment analysis demonstrated that nitazoxanide might be displayed anti-HCC effects by affecting gene targets implicated in multiple BP (peptidyl-tyrosine phosphorylation, protein autophosphorylation, protein phosphorylation, transmembrane receptor protein tyrosine kinase (RTKs) signaling pathway, positive regulation of MAP kinase activity, peptidyl-serine phosphorylation, etc.). Protein phosphorylation is vital for performing various activities such as biological processes, cellular localization, etc.; however, its aberrant regulation contributes to several conditions such as HCC, etc. (Liu Y. et al., 2021). MAP kinase regulates various cellular functions (apoptosis, proliferation, differentiation, etc.). MAP kinase activity is upregulated in the majority of malignancies with a high incidence rate, such as pancreatic cancer, non-small cell lung cancer, and particularly HCC (Li et al., 2021). RTKs are membrane-bound receptors necessary for cell function. By phosphorylating intracellular substrate proteins, they promote communication between cells. They govern cell proliferation, differentiation, metabolism, migration, etc., to maintain cellular homeostasis and are at the hub of intricate signaling networks. RTK mutations or aberrant activation are common causes of the development of cancers, including HCC (Sudhesh Dev et al., 2021). In addition, the targets implicated in the treatment of HCC with the nitazoxanide drug are involved in multiple CC, including the plasma membrane, cytoplasm, integral components of the plasma membrane, cytosol, cell surface, etc. Moreover, the results demonstrated that the targets by which nitazoxanide treats HCC are implicated in multiple MF, including transmembrane receptor protein tyrosine kinase activity, protein serine/threonine/tyrosine kinase activity, protein tyrosine kinase activity, kinase activity, ATP binding, etc.

The KEGG enrichment analysis revealed that the molecular pathways underlying the anti-HCC effects of the nitazoxanide drug might involve pathways in cancer, PI3K-Akt signaling pathway, MAPK signaling pathway, proteoglycans in cancer, EGFR tyrosine kinase inhibitor resistance, apoptosis, hepatitis B, ErbB signaling pathway, microRNAs in cancer, etc. In spite of the fact that the PI3K–AKT signaling pathway regulates a wide range of cellular processes, its abnormal activation promotes the development of HCC (Rahmani et al., 2020). Consequently, PI3K-AKT suppression may be an alternative HCC therapeutic modality. RTKs trigger activation of the MAPK signaling pathway. However, its inappropriate modulation leads to abnormal cellular activity, including enhanced cell growth and proliferation, dedifferentiation, and survival, which are all implicated in the etiology of malignancies, including HCC (Delire and Stärkel, 2015; Moon and Ro, 2021). The upregulation of proteoglycans such as glypican-3 leads to the development of melanoma. However, HCC patients had the highest proportion of positive instances (Ahrens et al., 2020). Therefore, inhibiting proteoglycans may be a potential treatment option for treating HCC. Moreover, the network results of core targets and pathways demonstrated nine major pathways (Figure 10). Among nine major pathways, the top two pathways were pathways in cancer (degree 7) and proteoglycans in cancer (degree 7). Seven anti-HCC core targets (CASP3, EGFR, ERBB2, mTOR, MMP9, HIF1A, and PPARG) followed the pathways in cancer. On the other hand, SRC, CASP3, EGFR, ERBB2, mTOR, MMP9, and HIF1A followed the proteoglycans in cancer (Figure 9). Thus, these two pathways may significantly contribute to the anti-HCC effects of the nitazoxanide drug by modulating the expression of anti-HCC core targets.

To further validate the results, nitazoxanide’s impact on the eight anti-HCC core targets (SRC, EGFR, CASP3, MMP9, mTOR, HIF1A, ERBB2, and PPARG) was investigated in silico. The targets’ molecular interactions with nitazoxanide demonstrated efficient binding in docking studies. Together, our findings show that patients with HCC have elevated levels of transcriptional expression of the expected targets and that therapy with nitazoxanide may suppress the translational expression of those targets.

## Conclusion

In this study, we have successfully identified the anti-HCC core targets and their biological functions, molecular pathways, and the effect of nitazoxanide on HCC. The constructed network pharmacology revealed the significant interaction among the predicted targets that identified eight anti-HCC core targets (SRC, EGFR, CASP3, MMP9, mTOR, HIF1A, ERBB2, and PPARG) as the active bio targets of nitazoxanide in HCC. Furthermore, we found that nine key pathways are likely to be involved: pathways in cancer, proteoglycans in cancer, MicroRNAs in cancer, central carbon metabolism in cancer, lipid and atherosclerosis, Kaposi sarcoma-associated herpesvirus infection, ErbB signaling pathway, and EGFR tyrosine kinase inhibitor resistance, by which nitazoxanide treats HCC. Our study validates the notion that nitazoxanide’s anti-HCC effects may emerge from synergistic interactions across several targets and pathways, and our data offer evidence to support this notion. A molecular docking simulation demonstrated the potential for active interaction between the anti-HCC core targets and nitazoxanide. Our study provides a theoretical foundation for the idea that nitazoxanide may have unique therapeutic benefits in HCC, and the pharmacological targets that have been identified may be potential biomarkers in the treatment of HCC.

## Data Availability

The original contributions presented in the study are included in the article/Supplementary Materials, further inquiries can be directed to the corresponding authors.
